# Post-graduation career pathways: a nationwide survey among dental students in Germany

**DOI:** 10.1007/s00784-024-05535-3

**Published:** 2024-02-05

**Authors:** Daniel G. E. Thiem, Behrus Puladi, Lukas Seifert, Philipp Becker, Monika Bjelopavlovic, Patrick Magennis, Jörg Wiltfang, Felix Benjamin Warwas

**Affiliations:** 1 Department of Oral and Maxillofacial Surgery, University Medical Center Mainz, Augustusplatz 2, 55131 Mainz, Germany; 2 Department of Oral and Maxillofacial Surgery, University Hospital RWTH Aachen, Pauwelsstraße 30, 52074 Aachen, Germany; 3 Department of Oral, Maxillofacial and Facial Plastic Surgery, University Medical Center Frankfurt, Theodor-Stern-Kai 7, 60590 Frankfurt am Main, Germany; 4 Department of Oral and Maxillofacial Surgery, Federal Armed Forces Hospital, Rübenacherstr. 170, 56072 Koblenz, Germany; 5 Department of Prosthodontics, University Medical Center Mainz, Augustusplatz 2, 55131 Mainz, Germany; 6 Department of Oral & Maxillofacial Surgery, Aintree University Hospital NHS Foundation Trust, Fazakerley, Liverpool, UK; 7 Department of Oral- and Maxillofacial Surgery, University Hospital Schleswig-Holstein, Arnold-Heller-Straße 3/House 26, 241015 Kiel, Germany; 8 Department of Oral- and Maxillofacial Surgery, University Hospital Bonn, Sigmund-Freud-Straße, 53127 Bonn, Germany

**Keywords:** OMFS, Career aspirations, OMFS future, Dental students, Stereotypes

## Abstract

**Objectives:**

Oral and maxillofacial surgery (OMFS) has to compete with other specialties for the best candidates. With the upcoming change of generations (Z and Alpha) and the movement toward gender parity of dentistry, understanding changing preferences and misconceptions is essential.

**Material and methods:**

An online survey was conducted by the German-Association-of-Oral-and-Maxillofacial-Surgery (DGMKG) across German dental schools. The survey collected demographic data, academic background, and career aspirations, with a focus on OMFS. The dental student survey results were compared to a survey given to OMFS Specialists.

**Results:**

637 dental students, mainly female (70%), from 30 German universities participated. 27% had defined career aspirations post-graduation, with self-employment and academia being popular choices. 67% were unsure. Specializations leaned towards restorative dentistry (41%), orthodontics (36%), and prosthodontics (31%). While 73% showed interest in surgical practices, 20% were attracted in specializing in OMFS. Of those averse to OMFS, 78% cited long training duration as the deterrent, 12% were put off by perceived unattractive working hours. Other reasons included negative undergraduate experiences, scarcity of part-time positions, and perceived inadequate earnings.

**Conclusion:**

Accurate data is crucial for career decisions. OMFS societies must proactively share accurate information and guide students. OMFS offers family-friendly hours, and while its training might be longer than dental specialties, it is on par with other surgical professions.

**Clinical relevance:**

Dental students consistently regard OMFS as commendable career path. To guarantee sustained OMFS expertise, it is imperative to nurture this interest through dedicated academic mentorship and innovative education, thereby solidifying their professional direction.

## Introduction

Oral and Maxillofacial Surgery (OMFS), as well as other surgical specialties, are facing a new generation (mainly Z and in future Alpha) with different characteristics compared to Baby Boomers, Generation X or Millennials [1]. The gender balance in medicine and dentistry is also changing from male preponderance to gender parity [2]. For historical reasons, different countries have established distinct pathways and educational requirements for individuals who aspire to become OMFS. In almost all European nations, a medical degree is mandatory [3]. In the majority of European nations (20) including Austria, Belgium, Germany, Switzerland, and the United Kingdom dual medical and dental degrees and training are required [3–5]. In Germany OMFS is usually part of undergraduate dentistry courses but is rarely part of undergraduate medical studies. It is unsurprising that OMFS knowledge and competencies among medical students are less when compared to their dental counterparts. To promote recruitment to OMFS requires integration of the specialty within medical and dental curricula [6]. It will also imbue doctors specializing in various medical fields with a solid foundation in OMFS knowledge [7, 8]. In light of this, several potential solutions have been deliberated, including the integration of a second-degree program specifically tailored for interns within OMFS departments [9, 10].

Although dental and medical students have a wide range of career options, the OMFS dual degree is one of the most versatile specialties, covering a very broad range of practice. However, this requires a total training period of usually more than 16 years, even with shorter dental degrees integrated into the OMFS training.

To sustain students’ motivation towards embracing a career in OMFS, it is necessary to systematically gather overall demographic information and specific training-related data in OMFS across universities. This process also involves pinpointing influential elements such as personal training expectations, future aspirations, and a broader outlook within OMFS. Hence, the primary objective of this study was to investigate these aspects through a comprehensive nationwide survey targeted at dental students.

## Material and methods

An online survey was conducted by the board of the German Association of Oral and Maxillofacial Surgery – Deutsche Gesellschaft für Mund-, Kiefer- und Gesichtschirurgie (DGMKG) using a dynamic online questionnaire created in SurveyMonkey (San Mateo, California, USA). Depending on the participants answers, additional sub-questions were possible for further elaboration. Thus, participants were asked to answer 19 to 22 questions. The questionnaire was designed short to keep dropout rates as low as possible. Some questions were skippable. A pilot with internal validation of the questionnaires was performed by the authors. A concise overview over the contents of the questionnaire is displayed in Table 1. Subsequently, questions pertaining to study conditions, professional future plans, and various aspects of OMFS were posed. The final segment of the survey centered on the DGMKG. Participants were questioned about their awareness of DGMKG, their past attendance at DGMKG events, and if they would be interested in future student-centered events.

**Table 1:** Concise summary of the questionnaire’s content. The questions, including the conditions under which sub-questions are available, are outlined in columns. Sub-questions are distinguished by their italicized format

Questions	Condition for sub-questions
How old are you?	
Which gender are you? *a) Male* *b) Female* *c) Diverse*	
Which clinical semester are you studying in?	
Where do you study?	
What’s your semester’s student count?	
Dentistry is my [a) First degree, b) Second degree]	For each: If “Second degree”
*If second degree, what did you study before?*	
*If second degree, did you complete your first degree?*	
Are your parents [a) Dentists, b) Doctors, c) Neither nor]	
Have you already thought about your path after graduation?	For each: “Sure, everything already set in stone”
*Great, so you: (different options)*	
Have you already thought about your path after graduation?	For each: “I am not sure yet”
*Absolutely understandable, but do you tend to: (different options)*	
Do you want to be a surgeon in your future career?	
Do you want to become a maxillofacial surgeon?	For each: If “No”
*If no, what put you off?*	
	For each: If “Yes”
*What influenced your choice to pursue maxillofacial surgery?*	
*When you think of maxillofacial surgery, what do you find particularly fascinating (multiple answers possible)?*	
*How long have you wanted to become a maxillofacial surgeon?*	For each: If “Yes”
In which field of dentistry would you like to specialize?	
Have you ever heard of the German Society for Oral and Maxillofacial Surgery (DGMKG)?	
Have you ever attended an event of the German Society for Oral and Maxillofacial Surgery (DGMKG)?	
Would you be interested in DGMKG events for students?	

In total, students from all 30 German universities that offer dentistry as a degree program were contacted. In addition to the official emails from university deans’ offices, as channels to promote the survey. To amplify the impact, direct post links were made to the respective dental student councils on these platforms. Additional promotion was achieved through the printing and display of posters within the respective faculties, as well as the incorporation and projection of QR codes linked to the survey at the conclusion of lectures. Participants were invited to take part in this anonymized survey. The surveys were accessible between September 28th, 2022 and July 15th, 2023. In total, three postings on social media placed within 4 months after starting the survey. Given the conditional nature of this questionnaire, not all participants responded to all 22 questions. Therefore, the number of participants providing each response is presented as an absolute count, and additionally, the results are shown in parentheses as the percentage of total respondents to the particular question. In addition, a brief online survey of OMFS specialists was conducted to validate the responses of dental students with individuals actually working in the profession.

Results were collected using SurveyMonkey and analyzed using SPSS Statistics® (version 23.0.0.2, MacOS X; SPSS Inc., IBM Corporation, Armonk, NY, USA). Correlation analysis was done using Chi-squared and Z-test. *P*-values < 0.05 were considered significant and highlighted with an asterisk (*). Results were illustrated in Numbers (Apple, Cupertino, California, USA), Excel and Powerpoint for Mac (Microsoft, Redmont, Washington, USA).

## Results

### General data

**Fig. 1:**
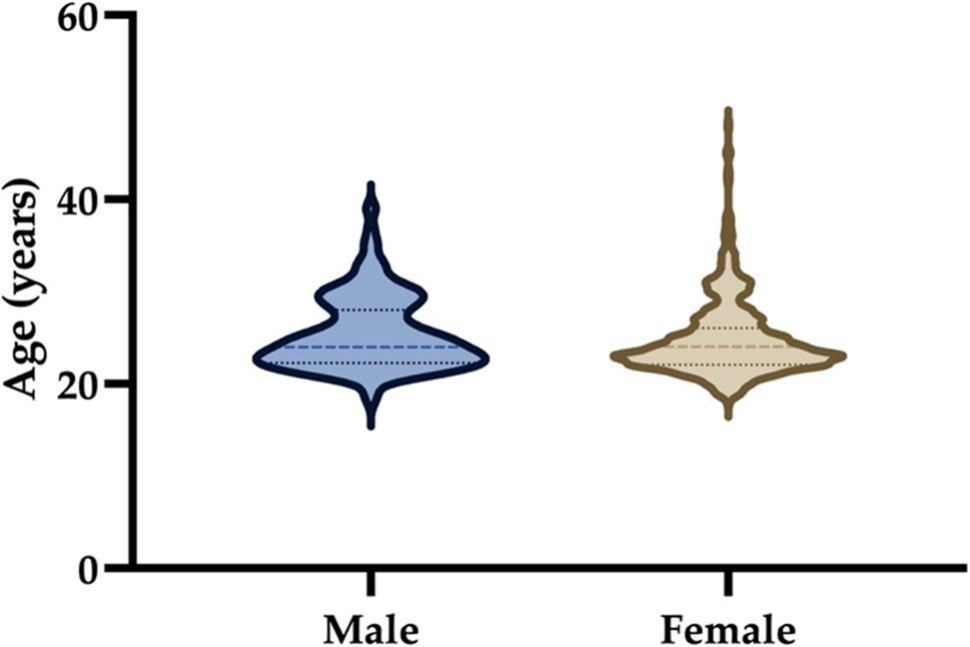
Violin plot shows the gender distribution of the participants in relation to the indicated age

In total, 637 participants took part in the survey, of which 634 individuals (99.5%) provided their age. The age range was between 18 and 48 years with an average age of 25 ± 4.2 years. 70.6% (*n* = 449) of participants were female, 29.1% (*n* = 185) male and 0.3% (*n* = 2) diverse (Fig. 1). The distribution of replies was not evenly spread across German Universities with 494 (77,5%) of 637 participants) originating from five (17%) of the total 30 institutions surveyed. Three universities did not contribute any responses.

**Fig. 2:**
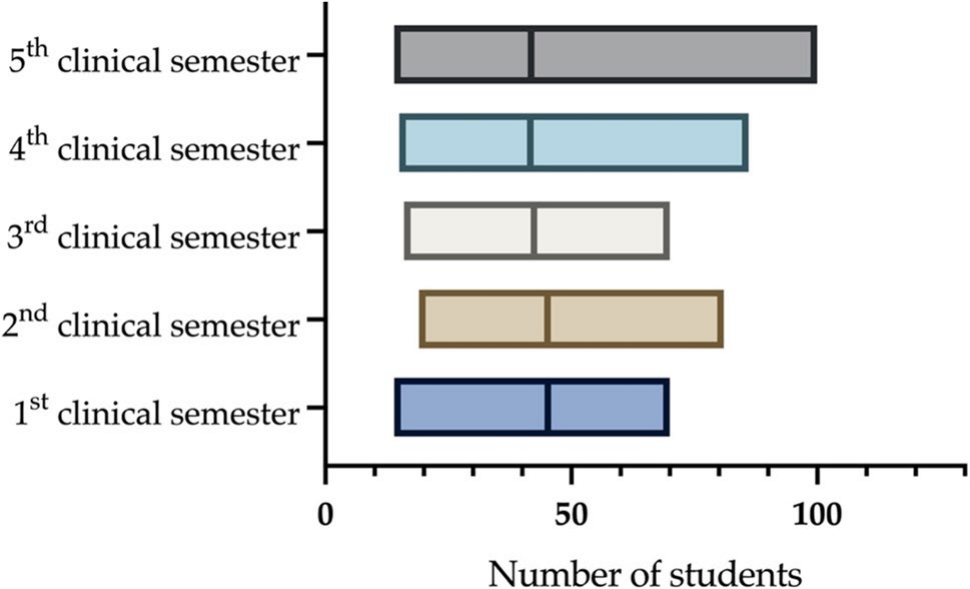
Floating bars (min to max) show the minimum, average and maximum number of students per clinical semester. The box’s vertical line signifies the mean, while its ends denote the minimum and maximum values

When stratified by year of study, the results demonstrated that 25.2% (*n* = 159/630) of respondents were in the first year, followed by 13.5% (*n* = 85/630) in the second, 14.6% (*n* = 92/630) in the third, 14.9% (*n* = 94/630) in the fourth, and 31.7% (*n* = 200/360) in the fifth and final year. On average, the first and second clinical years consisted of 45 students each, whereas the third, fourth, and fifth semesters each contained approximately 42 students (Fig. 2). Out of 635 respondents, 550 were pursuing their first degree in dental school, while 85 were in their second-degree program. 28/85 respondents (33%) in the second-degree program, had studied medicine as their first degree. Another 6 respondents (2.9%) studied chemistry, 11 (5.2%) enrolled in business administration, and 9 (10.6%) in biology. In addition, one person each (0.5%) had studied philosophy and history. Of the 85 participants, 31 (36.5%) had not studied any of the subjects offered for selection. Among 85 students on their second degree, 43 (50.6%) finished their first, with medical doctors comprising the majority at 23 (53.5%) (Table 2). Among the total of 637 respondents, 634 offered information pertaining to their parents’ occupational backgrounds. It was found that 15.1% (*n* = 96) of the student cohort indicated that at least one parent was professionally affiliated with the dental sector. A further 12.8% revealed parental involvement in the realm of human medicine. The remaining 457 students reported parents with no professional engagement in either dentistry or medicine.

**Table 2:** Cross-tabulation shows the distribution of first degree subjects chosen, as well as whether they were completed

		If second degree, what did you study before?							
		Biology	General medicine	Business administration	Chemistry	Physics	Philosophy	Neither/ Nor	Total
Did you complete your first degree?	Yes	2	23	4	0	1	0	13	43
	No	7	5	5	6	0	1	18	42
		9	28	9	6	1	1	31	85

### Postgraduate future

**Fig. 3:**
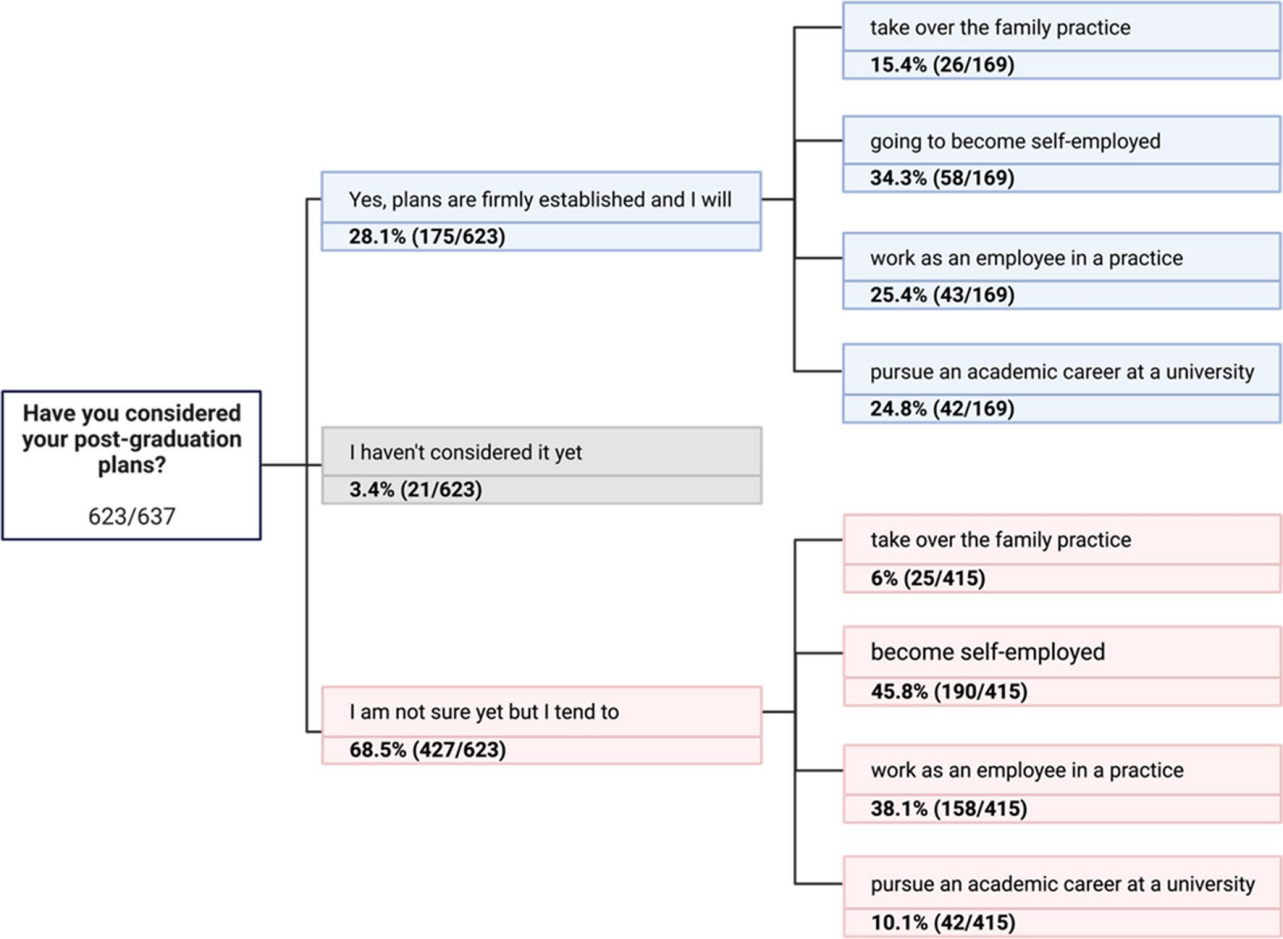
Tree diagram showing responses (percentage and total) to the question about post-university career

Regarding whether the students had already contemplated their post-graduation path, 28% (*n* = 175/623) responded affirmatively, indicating their plans were firmly established. The majority of participants, at 69% (*n* = 427/623), remained uncertain about their future trajectory. The smallest proportion, 3% (*n* = 21/623), had not yet given any thought to this subject. Among those who had already made a clear decision (169/623), 16% (27/169) intended to take over their family practice, 34% (58/169) planned to become self-employed, 25% (43/169) aimed to work as a dentist in an employed position, and 25% (42/169) were planning an academic career at a university. Of the students who were still uncertain, 6% (25/415) were likely to take over their parents’ practice, 47% (191/415) of the students tended toward self-employment, whereas 38% (158/415) would rather work in employment and only 10% (42/415) aspired to a university career (Fig. 3). Reviewing the future employment plans of those who have made a decision and gender, no significant differences were found. However, in the smaller group of students who were still undecided about their future career plans, there was a highly significant correlation between gender and the most likely intended work environment (X^2^ = 23.6; df = 3; *P* < 0.001). A detailed examination of the categorical relationships revealed that fewer women than expected opted for self-employment and/or taking over a parental practice, and more women chose to work in outpatient employment or pursue an academic career. Among male students, fewer than expected tended to work in outpatient employment or follow an academic career path. Conversely, a larger proportion of male students than expected planned to go into self-employment or take over a parental practice. The majority (73.1%; *n* = 444/607) of students indicated their intention to engage in surgical practice, however, the remaining 26.9% (*n* = 163/607) expressed no desire to pursue this path in the future. The question regarding which area of dentistry the students envisioned their professional future was answered by 292 students, with multiple selections allowed. Of these, 34% indicated a desire to specialize in dental surgery. Conversely, 41% of the respondents plan to specialize in restorative dentistry, followed by 36% in orthodontics, and 31% in prosthodontic dentistry.

### Career aspirations in oral and maxillofacial surgery

**Fig. 4:**
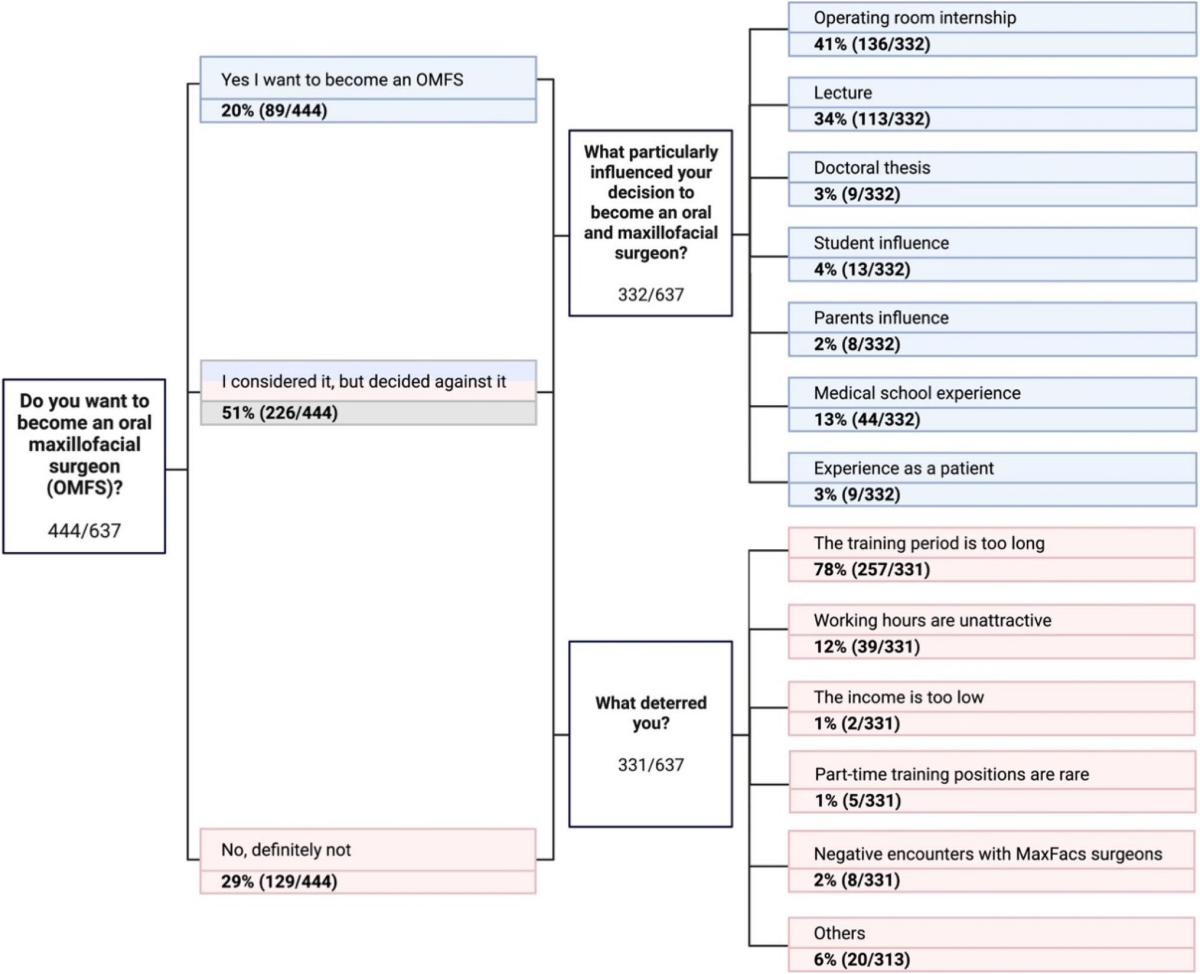
Tree diagram showing responses (percentage and total) to the question about ambitions to become an OMF surgeon

A total of 444 students responded to the question of whether they aspired to become OMFS surgeons. Of these, 20% (89/444) expressed a definitive desire to pursue a career OMFS. Only 29% (129/444) clearly negated and 51% (226/444) had at least considered it in the past but ultimately decided against it. In the realm of OMFS, the decision to specialize is often positively influenced by a variety of factors. According to our data, 41% of respondents attributed their choice for OMFS to the impact of their surgical internships during their dental undergraduate studies. This was closely followed by 34% who found the lectures or practical sessions during their dental medicine studies to be pivotal. A noteworthy 13.2% of respondents underscored the significance of their positive experiences during their practical year or a block internship within their medical studies as a decisive influence (Fig. 4).

**Fig. 5:**
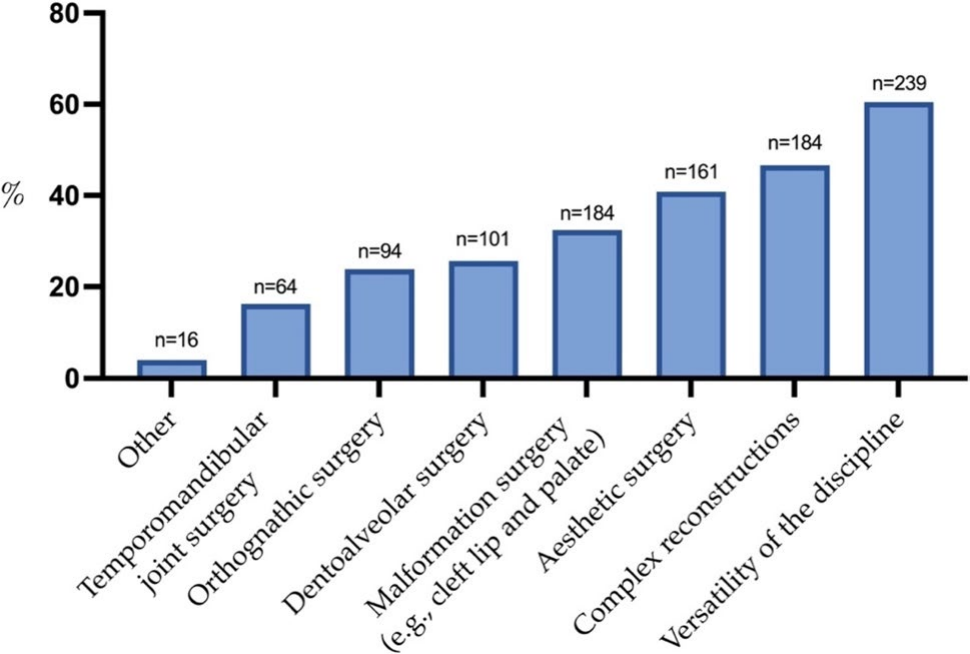
Bar chart showing which subfields of OMFS students find most fascinating

When asked when they had wanted to become OMFS surgeons, 81.4% (224 of 275) of the students answered "since dental school". Interestingly, 11.6% (32 of 275) had this career aspiration in mind since they were in high school, and another 6.9% since medical school. In this study, 60.5% of students found the versatility of OMFS most fascinating, followed by complex reconstructive surgery (46.6%) and aesthetic facial surgery (40.8%). Temporomandibular joint surgery was rated least fascinating, with 16.2% (Fig. 5). In evaluating the challenges associated with OMFS, 77.6% ranked the extended training duration as the primary concern, followed by 11.8% citing unfavorable working hours. The remaining 10.6% were distributed among the response options: a) other (6.1%), b) negative experiences with maxillofacial surgery (2.4%), c) insufficient part-time positions during training (1.5%), and d) inadequate income (0.6%).

### German society for oral and maxillofacial surgery

The German Society for Oral and Maxillofacial Surgery was known by 67% (186/278) of the students, with only 23/279 (8.2%) already having attended an event organized by the society. When asked whether there was interest in events organized by the German Society of Oral and Maxillofacial Surgery for students, 85.4% (*n* = 239/280) of respondents answered with yes. The remaining 41 respondents were not interested in such events.

### Viewpoint by specialists in oral and maxillofacial surgery

In this context, the assessment and viewpoints on individual career choices and initial motivations, as articulated by practicing OMFS specialists across various age brackets, are of particular relevance. Data pertaining to this were procured from a select cohort using the aforementioned and adapted questionnaire. In total, 23 OMFS specialists (20 male and 3 female) aged between 32 and 62 years (median age 40 years) responded to the questionnaire. Of these, 16 (70%) initially studied medicine, while 7 (30%) first pursued dentistry. Six (26%) survey participants had been specialists for less than one year, four for one year (17%), and two (9%) for three years. One participant (4%) had been active as an OMFS specialist for 5 years, four participants (17%) for over 5 years, two participants (9%) for over 10 years, and an additional four participants (17%) for more than 20 years. Regarding the nature of professional practice, 14 respondents (61%) indicated employment in a university hospital. Seven (30%) reported working in a private practice, while one participant (4%) was employed in a municipal hospital and another (4%) in an otherwise undefined setting. Of the 23 respondents, 15 (65%) had children, while the remaining 8 (35%) were childless. In response to the inquiry as to whether their career aspirations had been firmly crystallized during the period of dental medicine studies, 61% affirmed, while 39% negated. Among the participants who already had a career trajectory in mind, 30% aspired to self-employment during their dental medicine studies, another 30% aimed to work as hospital employees, and 30% sought to pursue an academic career. Additionally, one participant intended to transition into private practice after specialty training in a hospital, while another aimed to become head of department. Indeed, all participants with initial academic ambitions, along with 57% (4/7) targeting hospital employment, are currently situated in university hospitals. Conversely, 57% (4/7) of those aiming for private practice have successfully established themselves as such, while the remaining work in a university hospital. Among respondents, 17/23 (81%) expressed an initial intent to specialize in OMFS, while 19% arrived at this decision in the latter stages of their primary academic curriculum. When queried about which specialty they would choose today if they were limited to dental medicine, 11/22 (50%) opted for oral surgery, 9/22 (41%) for orthodontics, and 2/22 (9%) chose conservative dentistry. In their decision to specialize in OMFS, 54% (12/22) highlighted surgical internships in dental studies, 41% (9/22) noted medical study experiences, 27% (6/22) pointed to influential lectures, 23% (5/22) to doctoral work, 18% (4/22) to parental guidance, and 5% (1/22) to peer influence.

When queried about their primary interest (multiple selection possible) in OMFS, 70% (16/23) emphasized the field’s overall versatility, 48% (11/23) each selected orthognathic, complex reconstructive, and aesthetic surgery, 39% (9/23) chose craniofacial anomaly surgery, 22% (5/23) dentoalveolar surgery, and 13% (3/23) temporomandibular joint surgery. In gauging areas believed to particularly captivate students, 87% (20/23) cited complex reconstructive surgery, 39% (9/23) craniofacial anomaly surgery, 35% (8/23) orthognathic and aesthetic surgery, 30% (7/23) general versatility, 9% (2/23) dentoalveolar surgery, and 4% (1/23) temporomandibular joint surgery. In response to the proposition that the duration of OMFS training is prohibitively extensive, 52% (12/23) of the surveyed OMFS specialists concurred, whereas 26% (6/23) disagreed. The remaining respondents remained neutral on this particular point. Concerning the notion that reduced earnings deter the choice of OMFS as a specialty, 35% (8/23) of OMFS specialists agreed, 56% (13/23) disagreed, and the remaining 9% were neutral. Regarding the counterargument that insufficient part-time positions during training deter specialization in OMFS, 23% (5/22) of participants agreed, 50% (11/22) disagreed, while the remaining 27% (6/22) were neutral. Lastly, the surveyed OMFS specialists were queried on whether, from today’s perspective, they would choose OMFS again. A majority of 78% (18/23) affirmed this, whereas 22% (5/23) indicated they would not make the same choice again. Regarding recommending OMFS training to aspiring dental students, 61% (14/23) said yes, while 39% (9/23) answered with no.

## Discussion

For every profession, the acquisition and promotion of young talents is pivotal for its future existence. Therefore, a professional society’s essential task is to support and cherish each new generation. This is even more important when the profession is small and highly specialized. All this applies to OMFS. This survey provides insight into the career plans of young dental students and the mechanisms and preferences that lead them to their decisions. The gathered data offers insights into the aspirations and concerns of the upcoming generation of dentists. Addressing these is essential for the strategic recruitment of young professionals, ensuring the competitiveness of the OMFS specialty.

The survey results highlight that over 70% of students lack a definitive future plan. Conversely, 96.6% are contemplating further professional training and career planning during their studies, indicating that students are receptive to guidance in shaping their future trajectories. Contrary to what is sometimes commonly claimed, the proportion of students with a medical or dental family background was only about one-third (27.9%). The percentage of students whose parents are dentists was only 15.2%. However, more than half of these were determined to take over their parents’ practice or were at least considering it. The influence of parental practice on career planning is, therefore, vital, emphasizing how the perspectives and motivations of dental students differ significantly depending on cultural and social background [11].

A closer look at the participants’ career aspirations reveals that the most popular form of professional practice continues to be set up in their own practice. As already described in other studies [12], the proportion of those who want to pursue this path was around 50%. Of those who have already defined their career, the proportion of those who wish to take over their parents’ practice was almost three times higher. One-third of all students aspire to employment in a practice without self-employment, but only 14% to employment in a university hospital. The percentage of students who want to pursue an academic career was low. This finding is particularly notable because universities offer safe employment with comparatively high wages for those starting their careers. In addition, engagement at the university provides the opportunity to pursue research activities. It must raise the question of how the environment of university dentistry can be improved to make employment more attractive. Trottmann et al. have already made suggestions in their work on this topic, like formulation of clear, annually-reviewed tenure guidelines with specific milestones, identify job priorities, understand the importance of teaching, and seek mentorship or showcasing faculty successes to maintain enthusiasm, which are potentially suitable for adaptation to other systems [13].

### Duration of OMFS training program

Acquiring the knowledge and understanding of medicine and dentistry required to practice the full breadth and depth of the specialty of OMFS through dual degree training creates a long training pathway. This may be one of the reasons why the dental specialty of oral surgery is considered as an alternative (5 years of dentistry + 3 years of training) by some who do not want to follow this path in Germany. Although this allows to perform dentoalveolar procedures similar to a dual degree OMFS, it comes at the expense of most of the scope of practice.

Instead of turning to oral surgery, efforts should be made to improve the OMFS dual-degree training program. By applying the Bologna principles to reduce repetition of training between dental and medical training, this pathway can be shortened. For example, in Germany, dental graduates who studied medicine must repeat courses such as anatomy, biochemistry, or physiology, even though they have already taken them in dental school, because they are not considered equivalent. Medical graduates are required to take a general surgery exam in dental school, even though they took it extensively in medical school. Recognition from medicine to dentistry or vice versa often depends on the goodwill or location of the university. There are also examples across Europe of limiting the expenses of acquiring dual degrees by incorporating both degrees into OMFS training pathways [3]. When compared to other surgical specialties in the United Kingdom, the average age that OMFS specialists complete training is less than 2 years longer than other surgical specialties [14]. At present there is no European nation where dual degree OMFS training includes every mechanism possible to improve and shorten training. Only by understanding what ‘good’ could look like and applying that understanding in the context of the priorities of future trainees can recruitment and retention be maintained.

### Rewarding career and work-life-balance in OMFS

It is by placing the long training duration and high-demanding working environment of OMFS into the context of the rewarding career and work-life balance which will attract the best candidates to our specialty. OMFS specialists can work in diverse work environments, ranging from roles within (non-) university hospitals and private practices to academia, scientific research, and even military medical services [15, 16]. To ensure a thriving pipeline of dedicated professionals, OMFS must be compellingly presented as an enticing surgical specialty and career avenue for students and graduates. Clear positive information about OMFS care and careers should be present within the current dental and medical curricula. Where the curriculum is too full, this must be delivered with effective extra-curricula programmes ensuring that OMFS is not overshadowed by other specialties [17, 18]. Consequently, for the field of OMFS, active involvement and collaborative design of educational content during the initial stages of training for both dental and, notably, medical students is imperative.

### Surgically focused careers for dentists

The decision for future specialization is crucial within the surgical realms of OMFS. Encouragingly, 73.1% of students expressed a desire to engage in core surgical activities, emphasizing their keen interest in hands-on surgical procedures. This aligns with Marshall et al.’s study, which linked positive surgical placements to increased surgical career interest [19] and is supported by the present results on students attributing their interest in OMFS to positive lecture experiences and operating room internships. In accordance, Dhima et al. also demonstrated that joy and passion for the profession are the most significant factors influencing career planning [12]. Approximately one-third of those interested in surgery intended to specialize in the field of OMFS. It’s important to provide future general dentists with foundational skills and to support those aiming for OMFS specialization, highlighting the vital role of dedicated university surgical faculty. In this context, the significance of role models should not be underestimated, and the interaction with colleagues in the field of OMFS has a formative impact on students.

### Gender balance in surgical careers

Canadian studies have highlighted patient benefits with female surgeons [20, 21]. OMFS must ensure gender parity isn’t hindered by misconceptions about work hours or part-time roles. UK trends show significant gender shifts in OMFS [22]. Marti et al. found gender differences in OMFS perceptions, with female students reporting less favorable experiences, impacting their career interest [20]. Strong mentorship is crucial in OMFS, especially given the lengthy training [21]. Face-to-face mentorship is particularly effective for junior faculty advancement, more so than online lectures or self-study, and this is vital for recruiting female staff [22].

In this study, 70.6% of respondents were female, reflecting the broader trend of 67.1% female dental students in Germany [23]. Career planning shows gender disparities [24]. Male students often choose self-employment, while female students lean towards employment. This suggests a future rise in dentists seeking employment, considering the current low percentage of salaried positions [5]. Women’s lesser pursuit of surgical careers, as Sanfey et al. emphasize, is often due to perceived family-professional conflicts, necessitating adaptable work and training conditions [25].

### Working hours

European Working Time Directive (EWTD) is specific about the hours which can be worked in a single week [26]. These are defined based on safety models for patients and staff. In some nations in Europe, such as the UK, the EWTD has created a fundamental change in the culture within medicine in general and surgery in particular. This improvement in working hours have supported trainees of both genders, particularly those with families. The changes required to meet the EWTD have necessitated changes to surgical training to make it more efficient and effective. Over 75% of students had previously shown interest in specializing in OMFS. However, by the time of this survey, still one-third remained committed to pursuing it. Primary deterrents included the extensive training duration and demanding work hours. The perception regarding both the duration of training and working hours is corroborated by the majority (52% and 56%) of the surveyed OMFS specialists. Working hours, especially during residency, can accumulate to more than 100 h a week, self-study at home excluded. Despite the extended and demanding training, the number of practicing OMFS has steadily increased [7]. In this context, a recent survey by the German Association of Surgeons (Berufsverband der Deutschen Chirurgie) revealed that Germany is still struggling to implement the ETWD for surgeons.

### Aging workforce, Less Than Full Time (LTFT) working and Increasing demand for services

It is essential to consider that a substantial portion of the baby boomer generation will retire in the upcoming years [27]. Moreover, there’s a rising trend towards part-time positions among practicing physicians, leading the German head association of statutory health insurance physicians to project a 0.1% decline in overall healthcare provision, despite the overall physician increase [7]. This trend will inevitably affect the field of OMFS. Ensuring a sufficient influx of new professionals is a critical task for the future.

With an aging society, an increase in highly complex diseases within the field of OMFS is expected in the foreseeable future [20, 21]. Simultaneously, the public’s awareness of aesthetic procedures and the potential advancements in modern OMFS are on the rise [22]. Consequently, there is a projected demand for specialized expertise in this particular discipline. The prioritization of cultivating a new generation of highly skilled professionals becomes imperative to ensure the sustained provision of patient care over the long term. This appears to pose a challenge according to our findings, as the awareness of a healthy work-life balance is exceptionally high among the current generation of students [23].

### Engaging young dentists and doctors

The issue of recruiting young physicians has been previously addressed by other authors in the literature, where they have presented innovative approaches to mitigate potential negative associations with specific specialist groups [24, 28]. In this context, early engagement with specialist staff appears to yield positive outcomes. For instance, Hu et al. elucidated that the establishment of interest groups within the realm of otorhinolaryngology resulted in a favourable influx of medical students who had the opportunity to explore their interest in the specialty [25]. Consequently, they were seamlessly integrated into a consortium of researchers, surgeons, and decision-makers at an early stage. This integration allowed them to gain first-hand experience of clinical routines and the diverse nature of the specialty, as in the case of otolaryngology.

In a similar vein, initiating early interactions with students through elective courses, as well as incorporating professional field exploration into the curriculum under the new dental licensing regulations in Germany, would be a strategic measure. Such initiatives could capitalize on personal interactions with potential future colleagues, fostering the appeal of the OMFS profession. In 8 European countries the OMFS specialty is based on a single medical degree with some incorporated dental training, short of a dental degree [3]. Specialty training in Maxillofacial Surgery (basic medical training) is 6 years minimum compared to 5 years for dual degree OMFS. The trend in Europe is towards dual degree OMFS based on the perceived benefits to patients and surgeons (21 nations have mandated dual degree training). Creative solutions are needed to reduce the training duration for aspiring OMFS specialists. A noteworthy development in this regard is the introduction of the “run-through” program in 2014 within the UK which allowed trainees to complete 1 or 2 years of basic surgical training before joining a five-year OMFS programme. The UK also has a requirement that allows trainees to opt for Less than Full Time Training (LTFT) without needing to specify a reason. This approach supports female trainees to take a little longer to complete the training programme [29]. Flexible training enhances gender inclusivity within the field [30]. The recent restructuring of medical and dental licensing regulations offers an opportunity to explore innovative pathways. For the upcoming generation, aspects such as work-life balance and the compatibility of family and career are becoming increasingly crucial [31]. It may be more straightforward to enhance the appeal of the OMFS discipline through short-term measures addressing these concerns.

## Limitations

Results from our survey and previous literature must be interpreted cautiously. (1) First, the main proportion of participants was from only a small number of universities in Germany, leading to a selection bias. (2) Participation was free of choice, so students being more attracted to the topic of career planning and surgery may be overrepresented in this study. (3) Furthermore, the specialty of OMFS does not have a standardized training protocol internationally. In contrast, countries with single licensure for OMFS may have different tendencies toward pursuing a specialization in OMFS.

## Conclusions

Both medicine and dentistry are facing significant challenges. Societal shifts and the changing demographics of students render particularly time-consuming and high-responsibility professions less appealing. Especially high-demanding specialties like OMFS may be disproportionately affected. It is imperative to proactively address these concerns, listen to the younger generation, and not entirely disregard their aspirations. This entails striving for a balance between the constrained resources of the healthcare system and the expectations of younger generations. Long training hours still persist across Europe but, in nations which have adopted the European Working Time Directive and chosen to train smarter rather than longer, it appears that more can be done in less time. Surveys, such as the one presented, are an initial step toward guiding the specialty into the future. Based on such surveys, leaders of professional societies and politicians must evaluate potential measures to ensure that more young individuals, especially women, are attracted to pursue a career in OMFS. This implies the need to create opportunities that allow those individuals to pursue a professional career while simultaneously having and spending time with a family.

## References

[1] Schenarts PJ (2020). Now arriving: surgical trainees from generation Z. J Surg Educ.

[2] Al-Muharraqi MA (2020). Dental and medical dual qualification in Oral and maxillofacial surgery: a global identity. Br J Oral Maxillofac Surg.

[3] Magennis P, Hölzle F, Ulrich H-P, Acero J, Hutchison I (2022). The specialty of oral and maxillofacial surgery (OMFS) in Europe – part 2: training environment including the new Union of European Medical Specialists (UEMS) Oral and maxillofacial surgery european training requirement (OMFS ETR). Br J Oral Maxillofac Surg.

[4] European Union of Medical Specialists (2021) Training requirements for the specialty of oral & Maxillo-Facial Surgery (OMFS)1 European standards of postgraduate medical specialist training October 2021. European Union of Medical Specialists. https://www.uems.eu/__data/assets/pdf_file/0003/156045/UEMS-2021.36-European-Training-Requirements-in-OMFS.pdf. Accessed 2023

[5] Laskin DM (2008). The past, present, and future of oral and maxillofacial surgery. J Oral Maxillofac Surg.

[6] Seifert LB, Sterz J, Bender B, Sader R, Ruesseler M, Hoefer SH (2017). Undergraduate medical students need more training in craniomaxillofacial surgery: a comparative study between medical and dental students. Innov Surg Sci.

[7] Harris K, Jefferies C (2019). A multi-site cross-sectional survey exploring medical undergraduate knowledge of Oral and maxillofacial surgery. J Maxillofac Oral Surg.

[8] Mahalingam S, Kalia P, Mugilan S (2015). Oral and maxillofacial surgery in medical schools in the United Kingdom. Br J Oral Maxillofac Surg.

[9] Ilankovan V (2020). Training in the United Kingdom: are we fit for purpose?. Br J Oral Maxillofac Surg.

[10] Rapaport BHJ, Gill K, Douglas J, Ali T, Brown JS (2020). Training in oral and maxillofacial surgery: a medicine-first perspective. Br J Oral Maxillofac Surg.

[11] Karibe H, Kawakami T, Suzuki A, Warita S, Ogata K, Aoyagi K, Agholme MB, Dahllof G (2009). Career choice and attitudes towards dental education amongst dental students in Japan and Sweden. Eur J Dent Educ.

[12] Dhima M, Petropoulos VC, Han RK, Kinnunen T, Wright RF (2012). Dental students’ perceptions of dental specialties and factors influencing specialty and career choices. J Dent Educ.

[13] Trotman CA, Haden NK, Hendricson W (2007). Does the dental school work environment promote successful academic careers?. J Dent Educ.

[14] Douglas J, Begley A, Magennis P (2020). UK Oral and maxillofacial surgery trainees join the specialist list at a similar age to other surgical specialists. Br J Oral Maxillofac Surg.

[15] Brandt MT (2008). Transitioning from residency to private practice. Oral Maxillofac Surg Clin North Am.

[16] Bitonti DA, Steinle MA, Powers DB, MacKenzie TS, Lambert PM (2008). Oral and maxillofacial surgery careers in the military and Department of Veterans Affairs. Oral Maxillofac Surg Clin North Am.

[17] Kielty PGC, O’Connor BR, Cotter CJ, Goodson AMC, Payne KFB, Tahim A (2017). Medical students’ understanding of oral and maxillofacial surgery: an irish perspective. Br J Oral Maxillofac Surg.

[18] Seifert LB, Hoefer SH, Flammiger S, Russeler M, Thieringer F, Ehrenfeld M, Sader R (2018). A nationwide survey of undergraduate training in oral and maxillofacial surgery. Oral Maxillofac Surg.

[19] Marshall DC, Salciccioli JD, Walton SJ, Pitkin J, Shalhoub J, Malietzis G (2015). Medical student experience in surgery influences their career choices: a systematic review of the literature. J Surg Educ.

[20] Bojino A, Roccia F, Carlaw K, Aquilina P, Rae E, Laverick S, Romeo I, Iocca O, Copelli C, Sobrero F, Segura-Palleres I, Ganasouli D, Zanakis SN, de Oliveira Gorla LF, Pereira-Filho VA, Gallafassi D, Perez Faverani L, Alalawy H, Kamel M, Samieirad S, Jaisani MR, Rahman SA, Rahman T, Aladelusi T, Hassanein AG, Duran-Valles F, Bescos C, Goetzinger M, Bottini GB (2022). A multicentric prospective analysis of maxillofacial trauma in the elderly population. Dent Traumatol.

[21] Mady LJ, Nilsen ML, Johnson JT (2018). Head and neck cancer in the elderly: frailty, shared decisions, and avoidance of low value care. Clin Geriatr Med.

[22] Chen J, Chow A, Fadavi D, Long C, Sun AH, Cooney CM, Broderick KP (2021) The zoom boom: how video calling impacts attitudes towards aesthetic surgery in the COVID-19 era. Aesthet Surg J 41:NP2086-NP2093. 10.1093/asj/sjab274 10.1093/asj/sjab274 PMC8406860 34245237

[23] Harrison JL, Platia CL, Ferreira L, Soh M, Bugueno JM, Thompson TL, Quock RL, Finkelman M, Uzel NG (2022). Factors affecting dental students’ postgraduate plans: a multi-site study. J Dent Educ.

[24] Marti KC, Tishko G, Edwards SP, Inglehart MR (2021). Dental students’ OMFS-related experiences and interest in OMFS careers: an exploration. J Dent Educ.

[25] Hu A (2020). Reflections: starting an otolaryngology medical student interest group. Otolaryngol Head Neck Surg.

[26] The European Parliament and the Council (2003) Directive 2003/88/EC of the European parliament and of the council of 4 November 2003 concerning certain aspects of the organisation of working time. Official Journal of the European Union. https://eur-lex.europa.eu/eli/dir/2003/88/oj. Accessed 2023

[27] Magennis P, Begley A, Dhariwal DK, Smith A, Hutchison I (2022). Oral and maxillofacial surgery (OMFS) consultant workforce in the UK: reducing consultant numbers resulting from recruitment issues, pension pressures, changing job-plans, and demographics when combined with the COVID backlog in elective surgery, requires urgent action. Br J Oral Maxillofac Surg.

[28] Jarosz KF, Ziccardi VB, Aziz SR, Sue-Jiang S (2013). Dental student perceptions of oral and maxillofacial surgery as a specialty. J Oral Maxillofac Surg.

[29] Magennis P, Begley A, Douglas J, Dhariwal DK (2020). Duration of specialty training in Oral and maxillofacial surgery in the United Kingdom for trainees joining the OMFS specialist list between 2002 and 2019. Br J Oral Maxillofac Surg.

[30] Garg M, Collyer J, Dhariwal D (2018). “Run-through” training at specialist training year 1 and uncoupled core surgical training for oral and maxillofacial surgery in the United Kingdom: a snapshot survey. Br J Oral Maxillofac Surg.

[31] Scarbecz M, Ross JA (2002). Gender differences in first-year dental students’ motivation to attend dental school. J Dent Educ.

